# Native Valve Endocarditis Due to *Trichosporon mycotoxinivorans*—An Uncommon Presentation

**DOI:** 10.3390/jof12060447

**Published:** 2026-06-19

**Authors:** Kirun Gopal, Nandita Shashindran, Rajesh Jose, Praveen Kerala Varma

**Affiliations:** 1 Department of Cardiovascular & Thoracic Surgery, Amrita Institute of Medical Sciences and Research Center, Amrita Vishwa Vidyapeetham (Amrita University), Kochi 682041, India; rajeshj21304@aims.amrita.edu (R.J.); varmapk@gmail.com (P.K.V.); 2 Department of Microbiology, Amrita Institute of Medical Sciences and Research Center, Amrita Vishwa Vidyapeetham (Amrita University), Kochi 682041, India; nanditas@aims.amrita.edu

**Keywords:** *Trichosporon*, fungal endocarditis, native valve endocarditis

## Abstract

*Trichosporon* is a type of non-candida yeast-like fungus. At one time, it was commonly reported in immunocompromised patients, but after the introduction of fluconazole as prophylaxis and for the treatment of fungal infections, there was a decrease in the incidence of the disease. With the introduction of echinocandins as the first line of treatment for fungal infections, and the intrinsic resistance of *Trichosporon* to the drug, there has been a small but increased reported incidence of the disease. *Trichosporon* usually causes skin infections, but invasive disease can occur in vulnerable patients. Endocarditis due to *Trichosporon* has been reported rarely, and usually occurs in prosthetic valves. In this paper, we report a patient with dialysis-dependent chronic kidney disease who presented with fever and was found to have native aortic valve endocarditis. In view of the large vegetation, he underwent early aortic valve replacement. Both the blood and tissue cultures grew *Trichosporon* spp. Post-operatively, he developed fungal septic shock, deteriorated, and died. Invasive *Trichosporon* disease has been associated with high rates of mortality ranging from 30 to 90%. There is limited literature on endocarditis resulting from *Trichosporon*. Specific treatment recommendations are unavailable, and a combination of surgery and prolonged antifungal medication will generally be required.

## 1. Introduction

*Trichosporon* spp. are a type of non-candida yeast found in nature, in soil, decomposed wood, bird droppings, food, and water [1]. They have also been found as commensals in humans in the gastrointestinal tract, hair, skin, and vagina [2]. Though most initial isolations of *Trichosporon* were as a colonizer or as a cutaneous infection, evidence of invasive infection has also been found in vulnerable populations like immunocompromised patients, especially in patients with hematological malignancies. Transplant recipients, patients who received prolonged antibiotics, patients with indwelling catheters or prosthetic heart valves, and patients with chronic kidney disease have also been found to be susceptible to invasive infection. The genus *Trichosporon* is distinguished by the presence of arthroconidia, which are asexual propagules that disarticulate from true hyphae in urease-positive yeast [2,3]. The word *Trichosporon* is derived from Greek, with Trichos meaning hair and sporon meaning spores. This is related to its first identification as an infection presenting as nodules on body hair, which was called white Piedra. After *Candida*, *Trichosporon* infection is the second most common cause of yeast infection in humans. Fifty species of the genus *Trichosporon* have been described, of which six of the most clinical relevance are *T. asahii*, *T. asteroides*, *T. cutaneum*, *T. inkin*, *T. mucoides*, and *T. ovoides* [4]. Among these, *T. asahii*, *T. asteroides*, and *T. mucoides* have been associated with invasive infection. In the 1980s, trichosporonosis was the second most common fungal infection in patients with hematological malignancies [5]. Subsequently, with the widespread introduction of fluconazole as both prophylaxis and for the treatment of fungal infections, there was a sharp decline in the incidence of *Trichosporon* infection [6]. However, with the use of echinocandins as a replacement for the more toxic amphotericin B as the initial treatment of choice for suspected fungal infections, a breakthrough in fungemia due to *Trichosporon* has been described due to the resistance of *Trichosporon* to echinocandins [7,8]. *Trichosporon* rarely causes endocarditis, and what is reported in the literature is usually prosthetic-valve-related endocarditis. This patient presented with native valve endocarditis and usually *Trichosporon* would not be considered in the differential. This report highlights the possibility of the re-emergence of this pathogen as a cause of fungal endocarditis.

## 2. Case Report

**Figure 1:**
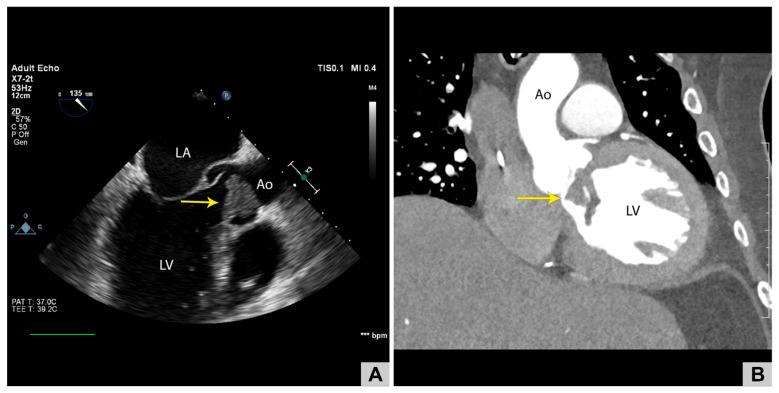
(**A**) Echo image of large vegetation in left ventricular outflow tract arising from the aortic valve. LA—left atrium. LV—Left ventricle. Ao—Aorta. (**B**) CT image of vegetation in left ventricular outflow tract. Ao—Aorta. LV—Left ventricle.

**Figure 2:**
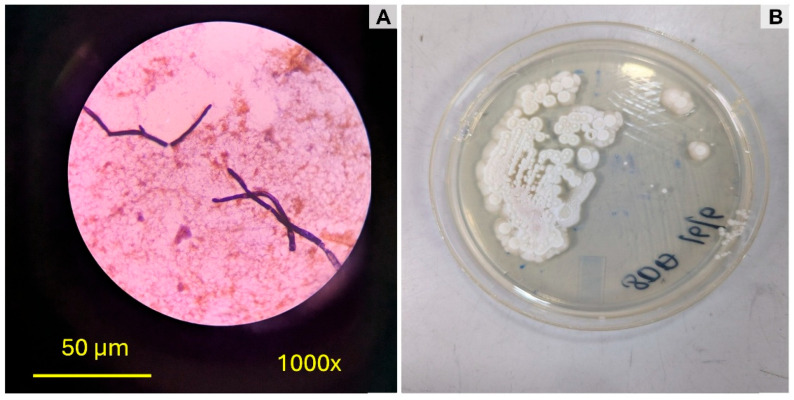
(**A**) Gram stain from blood culture showing filamentous fungal hyphae. (**B**) Dry white cerebriform colonies on Sabourauds dextrose agar after 48 h incubation.

**Figure 3:**
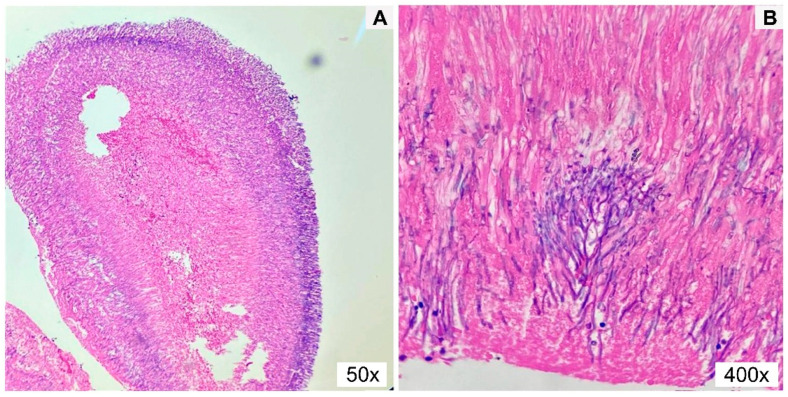
(**A**) Histopathology section from valvular vegetation with hematoxylin and eosin stain showing large fungal colonies. (**B**) Magnified histopathology section from vegetation with hematoxylin and eosin stain showing branched and septate fungal hyphae.

A 43-year-old male presented with a 3-day history of fever and breathlessness. He had a background of diabetes mellitus and dialysis-dependent renal failure. On examination, he was tachycardic, tachypneic, and febrile. Further evaluation by echocardiogram and computer tomography scan revealed a large aortic valve vegetation with significant aortic regurgitation (Figure 1A,B; Video S1). He was electively intubated and planned for early surgery in view of the large vegetation. Automated blood cultures were sent (Vitek2, bioMérieux, Marcy-l’Étoile, France) and he was started on ceftriaxone and vancomycin. The blood cultures were flagged as positive at 34 h of incubation, and the preliminary direct smear report indicated that yeast cells and pseudo hyphae were seen. Micafungin was added to cover the yeast since *Candida* is the most common cause of fungal endocarditis. Antigenic tests for fungi, β-D Glucan (6.83 ng/mL, ≥0.1 ng/mL positive), and galactomannan (2.08, ≥0.5 positive) from serum were both positive. The next day, he underwent surgical aortic valve replacement. A large vegetation attached to the right coronary cusp of the aortic valve was seen, with no involvement of the surrounding structures. The vegetation was sent for culture and histopathology (Figure 2 and Figure 3). The surgery was completed uneventfully, and he was shifted to the post-operative unit on a ventilator. Post-operatively, he did not become fully awake or oriented, with some spontaneous eye and limb movement but not to command. Following the procedure on day one, he developed high-grade fever and required increased vasopressor support to maintain blood pressure, and a diagnosis of post-operative vasoplegia or fungal sepsis was made. In the meantime, the blood culture and tissue culture grew identical dry, white, powdery colonies on sheep blood agar, chocolate agar, and Sabouraud dextrose agar within 48 h of incubation. The fungal isolate showed abundant arthroconidia on microscopy and was urease-positive. It also showed dry green colonies at 48 h of incubation on Candida HiCrome Agar (HiMedia, Thane, India). Vitek-2 identified *Trichosporon* spp. with low discrimination between *T. asahii* and *T. mucoides*. The isolate was found to have low MICs to voriconazole (Vitek-2 MIC ≤ 0.12 µg/mL) and fluconazole (2 µg/mL). The MIC of Amphotericin B was found to be 2 µg/mL, which is below the epidemiological cut-off value calculated by Francisco et al. for *Trichosporon asahii* [9]. The MICs of Caspofungin (>8 µg/mL) and micafungin (>8 µg/mL) were also high. Species identification by MALDI-TOF (Vitek MS, bioMérieux) was ordered, but the test failed to yield an identification, possibly because of the extremely dry consistency of the colonies. Subsequent Sanger sequencing of the isolate using primers targeting the D1/D2 region of the 26S rDNA identified it as *Trichosporon mycotoxinivorans* with 99.7% percentage identity. Currently, the MALDI-TOF (Vitek MS, bioMérieux) database does not include this pathogen. Voriconazole was initiated on post-operative day 2 and after 5 doses; trough levels were monitored, and the dose was titrated (target trough level 2 to 6 µg/mL). Despite treatment, the patient’s fever persisted, and blood pressure dropped with deterioration of neurological level and rising lactates, and the patient eventually expired on post-operative day 5.

## 3. Discussion

Fungal endocarditis is most commonly caused by Candida and Aspergillus, with Candida endocarditis being almost twice as common. Endocarditis due to *Trichosporon* has rarely been reported, with a recent article stating that there were only eight cases of *Trichosporon* endocarditis reported in the last 20 years [1]. *Trichosporon mycotoxinivorans* (current nomenclature *Apiotrichum mycotoxinivorans*) was first described in 2004 and is associated with infections in patients with cystic fibrosis [10]. Most of the reported cases of *Trichosporon* endocarditis have been prosthetic-valve-related, occurring in the immediate post-operative period to more than 10 years after surgery. Generally, patients with underlying medical conditions or patients with prior heart valve surgery with a prosthetic valve are more susceptible to the disease. Clinical presentation has been described as subacute with low-grade fever and leucocytosis, but our patient had a more acute presentation. In general, as with other cases of fungal endocarditis, vegetations are large and easily picked up on transthoracic echocardiogram, thus facilitating an earlier diagnosis. The large size of vegetation has also been implicated as the reason these patients can present more acutely with embolic events and early-onset heart failure. *Trichosporon* endocarditis has a poor prognosis, with the reported mortality being 56% in one review [4]. The reported incidence of embolism was also high, at 75%.

Diagnosis of *Trichosporon* endocarditis is enabled by the relative ease of growth in routine culture media used for bacterial blood culture, and there is no need for special fungal culture media. Cultures from valve tissue or vegetation are also usually positive. The Antigenic tests β-D glucan and galactomannan have been reported to be positive in *Trichosporon* infection, but are neither sensitive nor specific for the disease. One report of 33 patients with *Trichosporon* fungemia in which testing for β-D glucan was carried out reported 50% positivity [11]. Antigenic tests are more useful in this setting for their negative predictive value than for their positive predictive value. There is also a cross-reactivity with the cryptococcal capsular antigen glucuronoxylomannan. Therefore, the cryptococcal antigen detection test (latex agglutination or lateral flow assay) may be falsely positive in *Trichosporon* infections. One systematic review of invasive *Trichosporon* infections identified serum Cryptococcus glucuronoxylomannan (GXM) antigen in 4 out of 15 (26.6%) cases, while serum β-D glucan assay was positive in 9 out of 11 tested patients (81.8%) [8]. The source of *Trichosporon* in our patient is not clear. He had a known risk factor, dialysis-dependent renal failure, but he was being dialysed through a previously constructed arteriovenous fistula and not an indwelling catheter. *Trichosporon mycotoxinivorans* has been reported as a cause of fungemia in three patients who underwent hemodialysis, two of whom died despite receiving appropriate antifungal treatment [12]. Gut translocation, hospital-acquired infection, or the inherent risk of being a patient with renal failure were probably factors putting our patient at risk for infection.

Currently, the recommended treatment for *Trichosporon* infection is voriconazole or posaconazole as first-line treatment. However, there have been reports of the emergence of resistance to azoles due to the formation of biofilms [13]. Amphotericin B is considered as second-line treatment if Voriconazole fails. However, outcomes of Amphotericin B therapy on *Trichosporon* infection have generally not been as good compared to Voriconazole. Therapy may take the form of monotherapy or combined therapy, with good outcomes reported with Voriconazole alone. Speciation is of importance as there are differences in antifungal susceptibility between the species. A study from Taiwan on 267 *Trichosporon* isolates from clinical samples collected between 2008 and 2020 showed that *T. asahii* had significant higher MICs to fluconazole, voriconazole, isuvaconazole, and ravuconazole compared to other species. The study included only two isolates of *T. mycotoxinivorans*, which also interestingly showed high MICs to azoles [14]. Our patient’s infection proved resistant to Amphotericin B, and fluconazole MIC was relatively high. The treatment of *Trichosporon* endocarditis with drugs alone is probably insufficient. However, the number of reported cases is low, and there is no definite recommendation. Following the general recommendation for fungal endocarditis, surgery is probably required to allow the best possible chance for cure. Following surgery and 6 weeks of medical therapy, the duration of suppressive therapy in native valve endocarditis may be individualized based on the clinical picture and drug susceptibility testing; however, for prosthetic valve endocarditis, lifelong suppression may be required [3].

## 4. Conclusions

With the advances in medical care, we are seeing more patients with associated immunosuppression. This provides fertile ground for the emergence of newer infections in these susceptible patients. The standard treatment of Candida fungemia (which is the most common cause of fungemia) is echinocandins and Amphotericin B. However, the uniquely inherent resistance of *Trichosporon* to these drugs may lead to an increasing re-emergence of this fungus. Increased awareness of the infection, predisposing factors, and required treatment will hopefully lead to better outcomes for this life-threatening infection.

## Data Availability

The original contributions presented in this study are included in the article/Supplementary Material. Further inquiries can be directed to the corresponding author.

## Supplementary Materials

The following supporting information can be downloaded at: https://www.mdpi.com/article/10.3390/jof12060447/s1, Video S1: Echo loop showing large vegetation attached to aortic valve.
